# High Prevalence of Superficial Metastases Supports Percutaneous Ultrasound-Guided Biopsy for Diagnosis and Molecular Profiling of Advanced Lung Cancer: Results from a Prospective Cohort

**DOI:** 10.3390/cancers18142363

**Published:** 2026-07-22

**Authors:** Marta Viscuso, Vanina Livi, Giovanni Sotgiu, Valeria Cetoretta, Alessandra Cancellieri, Mariangela Puci, Angelo Minucci, Emilio Bria, Federico Cappuzzo, Silvia Novello, Rocco Trisolini

**Affiliations:** 1 Interventional Pulmonology Division, Fondazione Policlinico Universitario A. Gemelli IRCCS, 00168 Rome, Italy; marta.viscuso@gmail.com (M.V.); vanina.livi@policlinicogemelli.it (V.L.); 2 Clinical Epidemiology and Medical Statistics Unit, Department of Medicine, Surgery and Pharmacy, University of Sassari, 07100 Sassari, Italy; gsotgiu@uniss.it (G.S.);; 3 Department of Oncology, University of Turin, Azienda Ospedaliero-Universitaria San Luigi, 10126 Turin, Italy; vcetoretta@gmail.com (V.C.); silvia.novello@unito.it (S.N.); 4 Pathology Division, Fondazione Policlinico Universitario A. Gemelli IRCCS, 00168 Rome, Italy; alessandra.cancellieri@policlinicogemelli.it; 5 Molecular Diagnostics and Genomic Unit, Fondazione Policlinico Universitario A. Gemelli IRCCS, 00168 Rome, Italy; 6 Medical Oncology Division, Fondazione Policlinico Universitario A. Gemelli IRCCS, 00168 Rome, Italy; 7 Medical Oncology Division, Ospedale Isola Tiberina—Gemelli Isola, 00186 Rome, Italy; 8 Department of Translational Medicine and Surgery, Catholic University of the Sacred Heart, 00168 Rome, Italy; 9 Medical Oncology Division, IRCCS Regina Elena National Cancer Institute, 00144 Rome, Italy; 10 Department of Cardiovascular and Pulmonary Sciences, Catholic University of the Sacred Heart, 00168 Rome, Italy

**Keywords:** adenocarcinoma, endobronchial ultrasound-guided transbronchial needle aspiration, lung cancer, molecular profiling, ultrasound-guided biopsy

## Abstract

**Background:** Despite therapeutic advances, many patients with metastatic lung cancer still lack access to comprehensive molecular profiling, often due to inadequate biopsy samples. Percutaneous ultrasound-guided needle aspiration biopsy (US-NAB) from both lung and metastatic sites has shown promise in improving diagnostic and molecular profiling accuracy. However, data regarding its real-world utilization, diagnostic performance, and contribution to comprehensive molecular profiling in unselected patients with advanced lung cancer remain limited. **Methods:** We performed a secondary (post hoc) analysis of prospectively collected data from the Propheta Pro study, a prospective observational cohort of patients with advanced lung cancer. The aim of the present analysis was to assess patterns of invasive sampling procedures, with a particular focus on the prevalence of US-NAB utilization across histologic subtypes. We also assessed the diagnostic yield of US-NAB for cancer diagnosis, comprehensive genomic profiling, and PD-L1 expression. **Results:** Among the 348 patients enrolled, 123 (35.3%) underwent US-NAB, making it the most frequently utilized sampling technique overall and across individual histologic subtypes. Biopsy of metastatic sites was significantly more common than primary lung tumors (113, 91.9% versus 10, 8.1%; *p* < 0.001), with superficial metastases being the primary target (110, 89.4%). US-NAB demonstrated high diagnostic yields: 97% for histological diagnosis, 91.1% for comprehensive genomic profiling, and 95.8% for PD-L1 testing. Only two minor, self-limiting complications were observed. **Conclusions:** US-NAB is a highly effective yet underrecognized diagnostic option for advanced lung cancer, particularly given the high prevalence of accessible superficial metastases. Integrating US-NAB into interventional pulmonology services could enhance diagnostic and molecular profiling yields.

## Simple Summary

Many patients with advanced lung cancer require tissue sampling not only to establish a diagnosis but also to perform molecular testing that guides treatment selection. While bronchoscopy and CT-guided biopsy are widely used, ultrasound-guided needle aspiration biopsy (US-NAB) of superficial metastatic lesions remains underutilized despite being minimally invasive and readily available in many patients. In this prospective cohort, we found that more than one-third of patients underwent US-NAB, making it the most frequently used invasive sampling procedure. The technique achieved excellent diagnostic performance and provided tissue suitable for comprehensive genomic profiling and PD-L1 testing, with only two minor complications. Because superficial metastatic lesions were common in this population, greater integration of US-NAB into the diagnostic pathway may simplify tissue acquisition, improve access to molecular profiling, and reduce the need for more invasive procedures in appropriately selected patients.

## 1. Introduction

Most patients with lung cancer present with inoperable, advanced disease, where treatment focuses on optimizing quality of life, slowing disease progression, and prolonging overall survival. Clinical guidelines for advanced non-squamous non-small-cell lung cancer (NSCLC)—including the NCCN Clinical Practice Guidelines [1], the ESMO guidelines [2,3], and the Pan-Asian adapted guidelines [4]—recommend broad molecular diagnostic testing using next-generation sequencing (NGS) to identify actionable mutations for which targeted therapies exist. Additionally, these guidelines recommend determining tumor-derived programmed death-ligand 1 (PD-L1) expression using immunohistochemistry.

However, despite the availability of highly effective endoscopy- and CT-guided biopsy procedures, global patient access to NGS testing remains limited. Currently, fewer than 50% of patients undergo broad molecular testing due to various factors, including poor performance status that precludes biopsy, unfavorable tumor location, and inadequate biopsy sample quality [5,6,7,8,9]. The growing number of actionable genomic alterations and the increasing availability of biomarker-driven therapies have substantially increased tissue requirements for patients with advanced lung cancer. Consequently, obtaining adequate diagnostic material at the first invasive procedure has become a critical component of modern lung cancer care.

To address this gap, we integrated percutaneous ultrasound-guided needle aspiration biopsy (US-NAB) of primary tumors or metastatic sites in our interventional pulmonology program in late 2017. Although not yet included in major lung cancer clinical practice guidelines and infrequently performed by non-radiologists [1,2,3,4,10,11,12], US-NAB of primary tumors or metastatic sites has shown promise for improving diagnostic and molecular profiling success rates, while maintaining an excellent safety profile [13,14,15,16,17,18,19,20]. Moreover, because the procedure is typically performed without sedation, radiation exposure, or dedicated endoscopy or CT facilities, it may reduce procedural complexity and resource utilization in appropriately selected patients.

The aim of the present study was to assess the prevalence of US-NAB utilization and its contribution to histologic diagnosis and molecular profiling in a large cohort of patients with advanced lung cancer.

## 2. Methods

We performed a secondary (post hoc) analysis of data prospectively collected within the Propheta Pro cohort [20], a prospective observational study that enrolled consecutive adult patients (≥18 years) with suspected, previously untreated stage IV lung cancer identified by contrast-enhanced CT and/or PET, in whom tissue acquisition was required for histologic diagnosis and molecular profiling. Demographic, clinical, radiological, and procedural data—including invasive biopsy reports, final pathological diagnoses, and molecular profiling results—were systematically retrieved and analyzed.

For each patient, the interventional pulmonologist performing the procedure performed one or a combination of the following diagnostic sampling methods, selecting the least invasive procedure with the highest anticipated diagnostic yield according to imaging findings (whole-body contrast-enhanced CT and/or FDG PET): (1) HD videobronchoscopy for endoscopically visible abnormalities; (2) guided bronchoscopy for peripheral pulmonary lesions; (3) endosonography (EBUS-TBNA or EUS-B-FNA) from bronchoscopically invisible, peribronchial or peri-esophageal lesions; (4) percutaneous ultrasound-guided needle aspiration biopsy (US-NAB) from ultrasonographically visible pulmonary lesions with significant pleural contact, pleural lesions, or superficial lesions such as peripheral lymph nodes, muscular, subcutaneous or bone metastases; and (5) multiple procedures for patients requiring more than one of the above methods. Rapid on-site evaluation was available for each procedure.

The US-NAB procedures were performed by a team of 5 interventional pulmonologists. Each patient underwent an ultrasound examination using a Hitachi Aloka Arietta 850 ultrasound platform (FUJIFILM Healthcare Corporation, Tokyo, Japan), and the “target” lesion was evaluated with both a convex and a linear probe. When sampling was considered feasible and safe (i.e., absence of intervening large vessels), the skin was sterilized, and 2–5 mL of 2% lidocaine was injected in the subcutaneous tissue overlying the target lesion. The lesion was identified and sampled using a freehand technique with a 22-gauge needle (FARMAC-ZABBAN S.p.A., Calderara di Reno, Italy). The specimen obtained with the first needle pass was smeared on 2–4 glass slides. One slide was air-dried, stained with Diff-Quik and submitted to ROSE, whereas the remaining slides were put into ethanol solution and sent to the Pathology lab. Further sampling was guided by the ROSE results. If adequate sampling (i.e., representative lesional tissue or findings suggestive of a specific pathologic diagnosis) was confirmed by ROSE, 2 to 4 more needle passes were performed using a 16 G or 18 G cutting needle (Biomol biopsy set, Hospital Service SPA, Rome, Italy).

The primary endpoint was to determine the prevalence of US-NAB utilization in advanced lung cancer. Secondary endpoints included: (i) diagnostic yield of US-NAB; (ii) adequacy of US-NAB-derived samples for comprehensive genomic profiling and PD-L1 testing; (iii) complication rate; and (iv) prevalence of suspected “superficial” metastases, defined for the calculation of the present endpoint as lesions located in superficial layers of the body and visualized ultrasonographically with a linear probe.

In the absence of clinical signs or symptoms of complications, a focused ultrasonographic examination was performed approximately 1 h after the procedure to exclude perilesional hematoma and, when appropriate, pneumothorax was used before patient discharge. In addition, all patients were instructed to contact our unit should they develop signs or symptoms suggestive of complications within 48–72 h after the procedure.

Comprehensive genomic profiling was performed using TSO500HT^®^ (Illumina Inc., San Diego, CA, USA) or Oncomine Focus Assay^®^ (Thermo Fisher Scientific, Waltham, MA, USA) + Archer’s FusionPlex Lung Panel (Integrated DNA Technologies Inc., Coralville, IA, USA), depending on the quantity of extracted nucleic acids [21]. PD-L1 expression was assessed using the PD-L1 IHC 22C3 pharmDx kit on the Dako ASL48 platform (Dako, Glostrup, Denmark). These tests were assessed when clinically indicated and requested by the treating medical oncologist.

Data were summarized with absolute and relative (percentage) frequencies, as well as means (standard deviations) or medians (interquartile ranges), depending on data distribution. Point and interval (95% confidence intervals) prevalence estimates were calculated. Group comparisons of qualitative variables were conducted using chi-squared or Fisher’s exact tests, whereas comparisons of quantitative variables were performed using Student’s *t*-test or the Mann–Whitney U test, depending on parametric or non-parametric distribution. A two-tailed *p*-value of less than 0.05 was considered statistically significant. Statistical analyses were performed using STATA version 18 (StatsCorp, College Station, TX, USA).

## 3. Results

Table 1 summarizes the main characteristics of the 348 enrolled patients. The cohort was predominantly male (199, 57.2%) and comprised mainly ever-smokers (297, 85.3%), with adenocarcinoma as the most common subtype (248, 71%). Potential superficial metastatic targets amenable to US-NAB were identified in 110 patients (31.6%). The most frequent genomic alterations were *KRAS* mutations (29.5%), followed by *EGFR* mutations (11.8%) and *ALK* rearrangements (8.7%).

**Figure 1:**
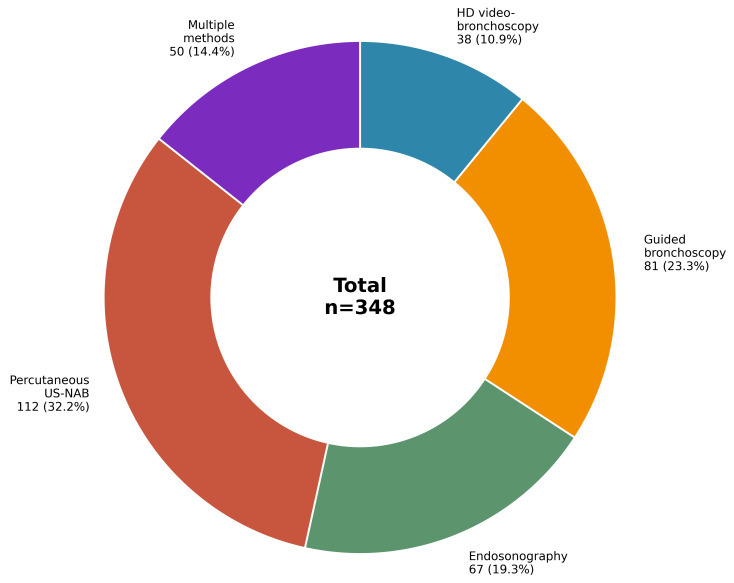
Pie chart showing the distribution of sampling procedures in the overall cohort (per-patient analysis). Values are presented as n (%).

US-NAB was performed in 123 of the 348 patients (35.3%), making it the most frequently used diagnostic procedure in the study cohort (Figure 1, Table 1).

**Figure 2:**
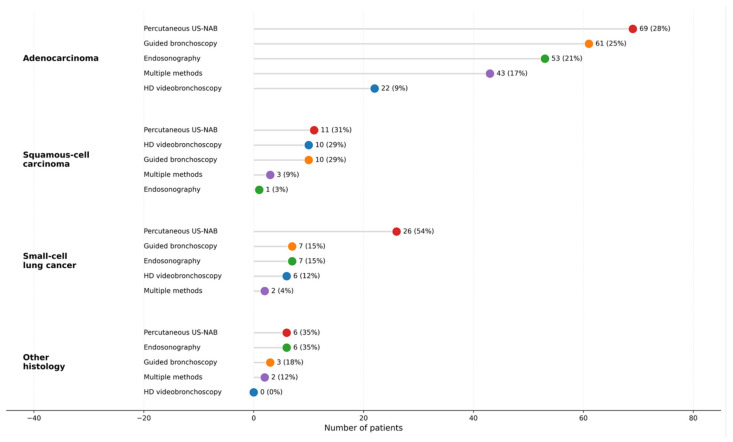
Cleveland dot plot depicting the distribution of sampling procedures across different lung cancer histologic subtypes. Values are presented as n (%) within each histologic subtype.

It served as the sole sampling modality in 112 patients (32.2%) and was combined with other sampling procedures in 11 patients (3.1%). Utilization of US-NAB varied across histologic subtypes and was highest in small-cell lung cancer, in which it was performed in 26 of 48 patients (54.2%) (Figure 2).

**Figure 3:**
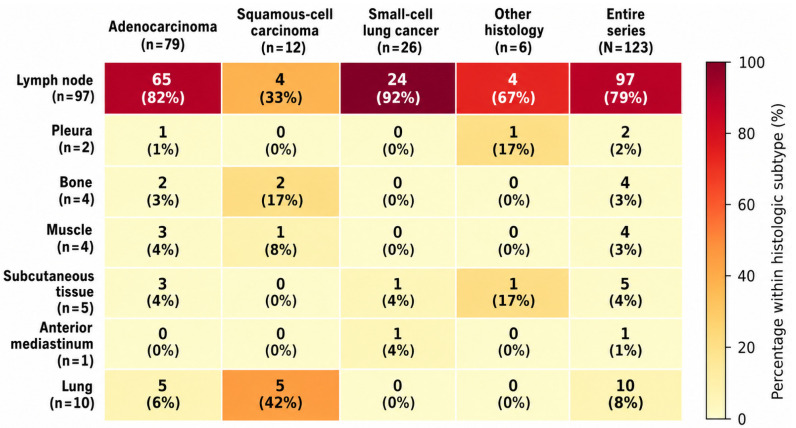
Heatmap illustrating the distribution of US-NAB target sites in the overall cohort and across different lung cancer histologic subtypes. Values are presented as n (%). Percentages are calculated within each column.

**Figure 4:**
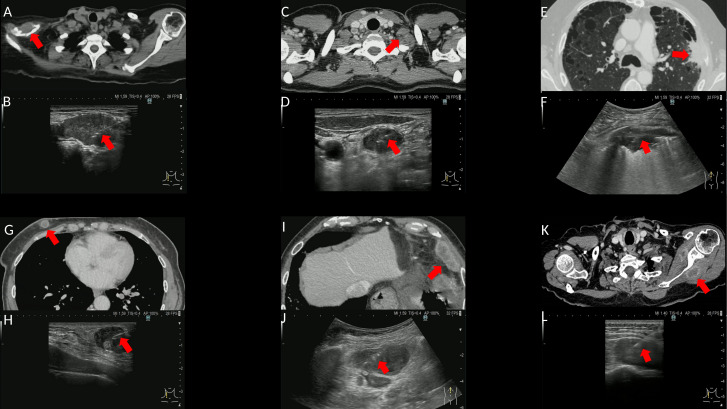
Axial CT images and corresponding US-NAB images of various metastatic and primary lung lesions. Panels (**A**,**B**): bone metastasis (arrows); Panels (**C**,**D**): supraclavicular lymph node metastasis (arrows); Panels (**E**,**F**): pulmonary primary tumor (arrows); Panels (**G**,**H**): subcutaneous nodular metastasis (arrows); Panels (**I**,**J**): pleural metastasis (arrows); Panels (**K**,**L**): periscapular muscular metastasis (arrows).

Among patients undergoing US-NAB, metastatic lesions were sampled far more frequently than primary lung tumors (113, 91.9% versus 10, 8.1%; *p* < 0.001) (Figure 3). Superficial metastatic sites accounted for the vast majority of procedures (110 of 123, 89.4%), with peripheral lymph nodes—including cervical, supraclavicular, axillary, and mammary lymph nodes—representing the most common biopsy targets (Figure 3 and Figure 4).

US-NAB established a histological diagnosis in 120 of 123 patients, corresponding to a diagnostic yield of 97.6% (95% CI, 93.0–99.5%). Comprehensive genomic profiling by NGS was successful in 72 of 79 cases (91.1%; 95% CI, 82.6–96.4%), while PD-L1 expression was successfully assessed in 92 of 96 cases (95.8%; 95% CI, 89.6–98.8%).

Two minor procedure-related complications occurred among patients undergoing US-NAB: one episode of vasovagal syncope and one case of transient chest wall pain following rib biopsy. Both resolved spontaneously without intervention.

## 4. Discussion

The present study provides three clinically relevant observations. First, superficial metastatic targets suitable for US-NAB are common in patients with stage IV lung cancer. Second, US-NAB was the most frequently utilized invasive sampling procedure in our prospective cohort. Third, samples obtained through US-NAB consistently supported both comprehensive genomic profiling and PD-L1 assessment. Together, these findings suggest that US-NAB may represent an underrecognized first-line diagnostic strategy in appropriately selected patients with advanced lung cancer.

US-NAB achieved diagnostic yields for histological diagnosis and molecular profiling that are comparable to endoscopy- or CT-based biopsy techniques while offering a better safety profile [22,23,24,25,26,27]. Notably, we observed no severe complications in patients undergoing US-NAB, whereas a recent prospective study reported a 2.4% severe complication rate associated with advanced diagnostic bronchoscopy in a similar cohort [28]. The low complication rate with US-NAB is likely due to the inherent safety of percutaneous biopsies of superficial metastatic sites, which comprised most of our biopsy targets, compared with lung lesion biopsies [27,28,29]. Importantly, the few pulmonary lesions sampled by US-NAB represented primary lung tumors with extensive pleural contact rather than superficial metastatic lesions and should therefore be interpreted separately from the much larger group of superficial metastatic targets. Unlike biopsies of superficial metastases, transthoracic sampling of pulmonary lesions carries a different risk profile and was performed only in a small minority of patients.

Our findings should also be interpreted in the context of the ongoing evolution of precision oncology. As the number of actionable molecular alterations and biomarker-driven therapies continues to expand, obtaining sufficient high-quality tissue has become increasingly important. The high success rates observed for both NGS and PD-L1 testing suggest that US-NAB may contribute not only to establishing the diagnosis but also to facilitating timely treatment selection. This consideration is particularly relevant given the persistent gap between guideline recommendations and real-world implementation of comprehensive molecular profiling [5,6,7,8,9].

Superficial metastases were identified and biopsied in nearly one-third of our patients, though this may be an underestimate, as most were staged at the time of sampling using contrast-enhanced whole-body CT rather than PET, which is more sensitive for detecting metastases. In a recent study, Park et al. compared PET with CT for selecting biopsy sites in lung cancer diagnosis and found that patients who had already undergone PET were more likely to undergo biopsy of an extrapulmonary site compared to those who underwent PET later [30]. This may suggest that our use of CT in staging potentially missed some superficial metastases that PET could have detected, further supporting the utility of PET in identifying suitable biopsy targets [31].

In addition to its safety and high diagnostic yield, US-NAB offers several practical advantages that may translate into greater efficiency of the diagnostic pathway. The procedure usually does not require sedation, avoids ionizing radiation, can often be performed in an outpatient setting, and is feasible even in patients with poor performance status. These characteristics may reduce procedural complexity and resource utilization compared with more invasive endoscopic or CT-guided procedures [13,14,15,16,17,18,19,20,32], although formal cost-effectiveness studies are still lacking.

From a healthcare systems perspective, wider adoption of US-NAB has the potential to improve diagnostic efficiency by reducing the need for more complex procedures, shortening diagnostic pathways, and preserving bronchoscopy suite and radiology resources for patients without accessible superficial targets. Such an approach could be particularly valuable in healthcare environments facing increasing demands for molecular testing and limited procedural capacity. These potential organizational benefits remain speculative and should be confirmed in prospective multicenter studies incorporating formal economic evaluations.

Despite these benefits, awareness remains limited, and US-NAB is absent from many international oncological and thoracic society guidelines for lung cancer diagnostics [1,2,3,4,10,11,12]. In our study, this lack of recognition was evident, as many patients initially referred for bronchoscopy were managed instead with US-NAB after detailed imaging review that identified a potential superficial metastasis.

The main limitation of this study is its single-center design and the involvement of experienced operators, which may affect external generalizability. However, the learning curve for US-NAB—particularly for superficial metastatic sites—is relatively short. A study evaluating the performance characteristics and complications of US-NAB during its first two years of implementation reported excellent diagnostic yield and safety profiles [17]. In addition, the absence of PET imaging may have led to an underestimation of the prevalence of superficial targets.

## 5. Conclusions

Superficial metastatic lesions suitable for US-NAB sampling are common in patients with stage IV lung cancer. This technique achieves high success rates for diagnosis and molecular profiling, while reducing procedural complexity and procedural risks, with the potential to decrease healthcare resource utilization in appropriately selected patients. Greater recognition of this biopsy technique in future clinical practice guidelines issued by leading scientific societies could increase awareness and promote its broader adoption within the interventional pulmonology community.

## Figures and Tables

**Table 1:** Baseline characteristics of the study cohort.

Variable	n = 348
**Age (years), median IQR**	68 (62–75)
**Male sex, n (%)**	199 (57.2%)
**Smoking, n (%)**	
Current	146 (42.0%)
Former	151 (43.4%)
Never	51 (14.6%)
**ECOG PS, n (%)**	
0	123 (35.3%)
I	191 (54.9%)
II	32 (9.2%)
III	1 (0.3%)
IV	1 (0.3%)
**Imaging studies ^§^ (n %)**	
CE whole body CT	224 (64.4%)
FDG PET-CT	3 (0.8%)
CE whole body CT + FDG PET-CT	121 (34.8)
**Superficial metastases (n %)**	110 (31.6%)
**Histology, n (%)**	
Adenocarcinoma	248 (71.3%)
Squamous cell carcinoma	35 (10.0%)
Small-cell lung cancer	48 (13.8%)
“Other” histology ^π^	17 (4.9%)
**Sampling method, n (%)**	
HD videobronchoscopy	38 (10.9%)
Endosonography	67 (19.3%)
Guided bronchoscopy	81 (23.3%)
Percutaneous US-NAB	112 (32.2%)
Multiple methods *	50 (14.4%)
**Genomic alterations °, n (%)**	
EGFR	30 (11.8%)
KRAS	75 (29.5%)
ALK	22 (8.7%)
ROS1	3 (1.2%)
BRAF	15 (5.9%)
MET	19 (7.5%)
RET	8 (3.1%)
NTRK	0 (0.0%)
ERBB2	8 (3.1%)
**PD-L1 expression ^, n (%)**	
<1%	59 (21.2%)
1–49%	88 (31.7%)
>50%	131 (47.1%)

Abbreviations: PS: performance status; US-NAB: percutaneous ultrasound-guided needle aspiration biopsy. ^§^ Available at the time of sampling. ^π^ Includes a combination of less common primary lung tumors (e.g., large-cell neuroendocrine carcinoma, sarcomatoid carcinoma, carcinoid tumors, adenosquamous carcinoma). * Refers to the combination of two or more of the sampling methods in the same patient. ° Applicable to the 254 patients who were indicated for cancer genotyping and had tissue specimens suitable for the analysis. ^ Applicable to the 278 patients who were indicated for PD-L1 testing and had tissue specimens suitable for the analysis.

## Data Availability

The raw data used for the present study can be obtained from the corresponding author upon reasonable request.
